# Structural investigation of zymogenic and activated forms of human blood coagulation factor VIII: a computational molecular dynamics study

**DOI:** 10.1186/1472-6807-10-7

**Published:** 2010-02-25

**Authors:** Divi Venkateswarlu

**Affiliations:** 1 Department of Chemistry, 1601, E. Market Street, North Carolina A&T State University, Greensboro, NC 27411, USA

## Abstract

**Background:**

Human blood coagulation factor VIII (fVIII) is a large plasma glycoprotein with sequential domain arrangement in the order A1-a1-A2-a2-B-a3-A3-C1-C2. The A1, A2 and A3 domains are interconnected by long linker peptides (a1, a2 and a3) that possess the activation sites. Proteolysis of fVIII zymogen by thrombin or factor Xa results in the generation of the activated form (fVIIIa) which serves as a critical co-factor for factor IXa (fIXa) enzyme in the intrinsic coagulation pathway.

**Results:**

In our efforts to elucidate the structural differences between fVIII and fVIIIa, we developed the solution structural models of both forms, starting from an incomplete 3.7 Å X-ray crystal structure of fVIII zymogen, using explicit solvent MD simulations. The full assembly of B-domainless single-chain fVIII was built between the A1-A2 (Ala1-Arg740) and A3-C1-C2 (Ser1669-Tyr2332) domains. The structural dynamics of fVIII and fVIIIa, simulated for over 70 ns of time scale, enabled us to evaluate the integral motions of the multi-domain assembly of the co-factor and the possible coordination pattern of the functionally important calcium and copper ion binding in the protein.

**Conclusions:**

MD simulations predicted that the acidic linker peptide (a1) between the A1 and A2 domains is largely flexible and appears to mask the exposure of putative fIXa enzyme binding loop (Tyr555-Asp569) region in the A2 domain. The simulation of fVIIIa, generated from the zymogen structure, predicted that the linker peptide (a1) undergoes significant conformational reorganization upon activation by relocating completely to the A1-domain. The conformational transition led to the exposure of the Tyr555-Asp569 loop and the surrounding region in the A2 domain. While the proposed linker peptide conformation is predictive in nature and warrants further experimental validation, the observed conformational differences between the zymogen and activated forms may explain and support the large body of experimental data that implicated the critical importance of the cleavage of the peptide bond between the Arg372 and Ser373 residues for the full co-factor activity of fVIII.

## Background

**Figure 1:**
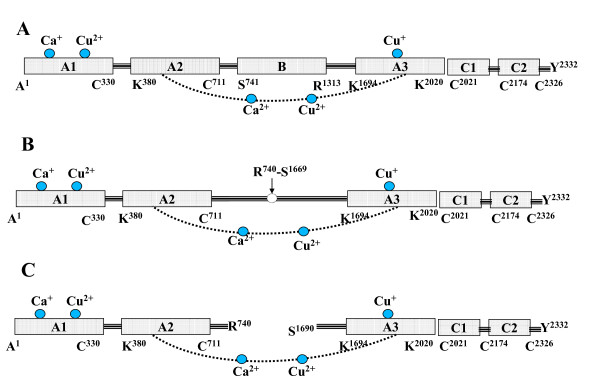
**The schematic representation of the sequence and domain arrangement of A) the full model of zymogen fVIII; B) the B-domainless fVIII, used in the present study; C) the activated fVIII**. The functionally important calcium and copper ions bound to the inter- and intra-domains are also shown.

The Human blood coagulation factor VIII (fVIII) is an essential protein involved in the intrinsic coagulation pathway. Factor VIII is synthesized as an ~300 kDa single chain protein containing 2332 residues and composed of six domains: A1-a1-A2-a2-B-a3-A3-C1-C2 [1]. The A-domains are interconnected by short linker peptides that are rich in clusters of acidic residues. The linker peptides correspond to the residues: 337-372 (a1) between A1 and A2 domains; 711-740 (a2) at the C-terminus of A2-domain and 1649-1689 (a3) between B and A3 domains. Activation of fVIII by thrombin or factor Xa (fXa) at three proteolytic sites Arg372-Ser373 (A1-A2 junction), Arg740-Ser741 (A2-B junction), and Arg1689-Ser1690 (B-A3 junction) yields functionally active factor VIII (fVIIIa) that circulates in blood as a hetero-trimer among the heavy chain domains A1, A2 and the contiguous light-chain domains A3-C1-C2. Factor VIIIa serves as a critical co-factor for proteinase enzyme factor IXa (fIXa) in the intrinsic pathway of blood coagulation cascade during the proteolytic activation of zymogen factor X (fX). The catalytic activity of fIXa itself is very poor in the proteolysis of fX, but increases dramatically when bound to the co-factor fVIIIa [2]. The role of fVIIIa is to increase the catalytic rate constant (K_cat_) of fX activation by several orders (10^6 ^fold) of magnitude and also to reduce the K_d _for its interaction with fIXa and the K_m _for substrate fX [3,4]. The clinical significance of fVIIIa is manifested by functional defects triggered by genetic mutations or acquired inhibitors that result in Hemophilia-A, a severe bleeding hereditary coagulation disorder affecting 1 in 5000 people [2]. The domain and sequence details of the models studied in the current work are shown in Figure 1.

The circulating fVIIIa in blood plasma has limited half-life due to rapid dissociation of A2 domain [5]. The loss of A2 domain has been attributed to the weak A2-subunit interactions with A1 and A3 domains [6]. Consequently, a number of experimental site-specific mutagenesis studies have been directed towards understanding the specific residues involved in the inter-domain interactions as well as its interactions with the enzyme fIXa and substrate fX [7-12]. Despite a great progress in such efforts, the atomic level details of the full structures of zymogenic and activated forms of fVIII still remain unclear. The detailed three-dimensional structures of fVIII and fVIIIa are essential pre-requisites not only to understand the structural features but also the interactions with its physiological activators (thrombin and fXa) and proteins associated with the intrinsinc tenase pathway (fIXa and fX). Recently two low-resolution (2R7E:3.7 Å and 3CDZ:3.98 Å) X-ray crystal structures of fVIII zymogen were reported [13,14]. Both models were derived by fitting the electron density space of fVIII crystals with the homology models of the individual domains derived from the human ceruloplasmin, bovine factor Va (fVa) crystal structure and a high-resolution X-ray crystal structure of C2 domain. The A-domains of fVIII are homologous to the copper-binding ceruloplasmin protein with ~35% sequence identity while the C-domains belong to discoidin family of proteins with variable homology. Both crystal structure models reported unusually high degree of thermal disorder with the average thermal B-factors of over 195 Å^2^. While these models provided a great detail about the overall global features of the domain organization within the X-ray crystallographic space, the low resolution of the crystal structures limits their reliability for the atomic-level structural details of the domain-domain interactions. Backbone superimposition of the two crystal structures (PDB: 2R7E and 3CDZ) revealed significant conformational differences in the solvent-exposed loop regions. Also, the reported crystallographic data lacks the structural information for the functionally important linker peptides that interconnect the A-domains. These regions include the acidic linker peptide between Pro333 (A1-domain) and Lys377 (A2 domain); the C-terminal peptide from Lys713 to Arg740 within the A2-domain.

In this paper, we developed the solvent-equilibrated B-domainless solution structures of zymogen fVIII and its activated form, fVIIIa, in several stages starting from one of the X-ray crystal structures (PDB ID:2R7E) [13] by refining the models using MD simulations in explicit water. The development of the full solution structure of fVIII zymogen followed the modeling and refinement of two key regions: 1) Refinement of the core region of the protein for which the crystal coordinates are available; 2) Prediction of the structures for two acidic rich linker peptides interconnecting the A1:A2 and A2:A3 domains for which the coordinates are not available. The solvent equilibrated solution structures of fVIII and fVIIIa predicted that the active and zymogenic forms may have significant conformational differences around the putative fIXa binding site in the A2 domain. While the proposed structure of the linker peptide between the A1 and A2 domains is predictive in nature and requires experimental validation, they corroborate well with the existing hypothesis that the exposure of the putative fIXa binding loop (Tyr555-Asp579) in the A2-domain is critical for productive interactions with fIXa enzyme during the intrinsic tenase (fVIIIa.fIXa) complex formation. A detailed account of the intra and inter-domain interactions among the five domains and a correlation with known biochemical mutagenesis data is presented.

## Results

**Figure 2:**
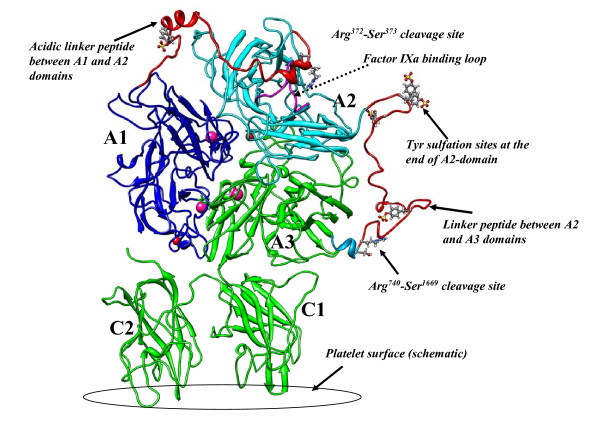
**The solution structural model of human B-domainless fVIII zymogen derived from the MD snapshot that corresponds to 75 ns of MD simulation trajectory**. The coordinating calcium (red spheres) and copper (pink spheres) ions, tyrosine sulfation sites (ball-and-stick) are highlighted. The A1-A2 acidic linker may be seen blocking the putative fIXa binding Y555-D569 loop (magenta color) in the A2-domain (orange color). The solvent-exposed proteolytic site (Arg372-Ser373) is also shown. The platelet surface shown in the figure is merely a schematic representation and not modeled in the current study.

**Figure 3:**
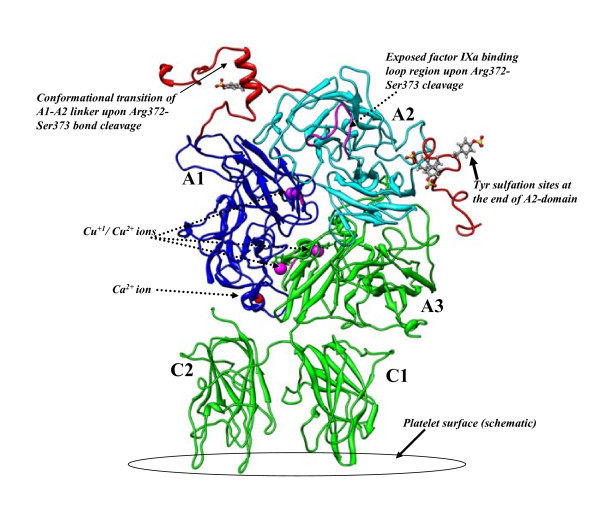
**The solution structure of activated fVIII derived from the MD snapshot that corresponds to 80 ns of MD simulation trajectory**. The conformational reorganization of the A1-A2 acidic linker peptide (orange color), upon the cleavage of the Arg372-Ser373 bond, may be seen from the MD simulations. The exposed fIXa binding loop in the A2-domain is highlighted (magenta color).

Human fVIII model in the current study was built using the 3.7 Å resolution X-ray crystal structure of fVIII published by Shen et al [13]. Another X-ray crystal structure with 3.97 Å resolution using similar synthetic construct and crystal form was published [14]. Both models were built based on fitting the homology models of fVIII into the electron density map of fVIII X-ray crystallographic space (PDB ID: 2R7E and 3CDZ). The alignment of the backbone atoms of the two structures revealed significant conformational differences in several solvent-exposed loop regions, though the core region of the both structures was similar with an overall RMS difference of 0.98 Å for the backbone atoms. The fIXa binding loop (Tyr555-Asp569) within the A2-domain and the surrounding region was also markedly different in both crystal structures. This region was implicated in several experimental studies as functionally important for fVIIIa:fIXa interactions as well as thrombin interactions with fVIII during the activation. The A1-A2-A3 domains of fVIII share ~35% sequence identity with the structure of human ceruloplasmin, from which the X-ray structural models were built. It is not surprising that such low sequence identity reflected on the poor stereo-chemical quality of the crystal structures. The analysis of the Ramachandran plot showed that the initial X-crystal structure has ~290 residues (out of 1329 residues) as the outliers in the Phi-Psi dihedral space (Additional file 1:Figure S1). In contrast, the MD equilibrated fVIII zymogen structure, corresponding to 70 ns of MD trajectory, showed marked improvement in the stereochemical quality with only three non-glycine residues (out of 1404) as the outliers in the Ramachandran plot. These residues are located in the solvent-exposed loop regions and some residues appeared to vary from one snapshot of MD trajectory to the other, perhaps representing the conformational sub-states. The overall structure of fVIII showed 99.7% of the residues in the generously allowed region of the Ramachandran plot. Similarly, the solution structure of fVIIIa showed 99.5% of the residues in the most favorable region with only four non-glycine residues in the outlier region. The evaluation of the "goodness of fit" of the model in comparison with the proteins in the crystal structure database by ProSA server [15]https://prosa.services.came.sbg.ac.at/prosa.php ranked the MD derived fVIII zymogen model with a Z-score of -11.8 and that of the fVIIIa structure with the corresponding score of -11.5. These values were considerably less than the maximum acceptable value of -0.3 when compared with the range of native conformations of the PDB templates. The corresponding Z-score of the X-ray structure was -7.4 (PDB = 2R7E). The domain-wise alignment of the starting X-ray crystal structure with the MD derived model showed overall agreement in the core-region of the domains but significant differences in the domain-domain interface in the A-domains. The superimposed structures are shown in Additional file 2: Figure S2. The structures of the fVIII zymogen and its activated forms, corresponding to 75 and 80 ns of MD snapshots respectively, are shown in Figures 2 and 3 with highlighting the important regions in the proteins.

**Figure 4:**
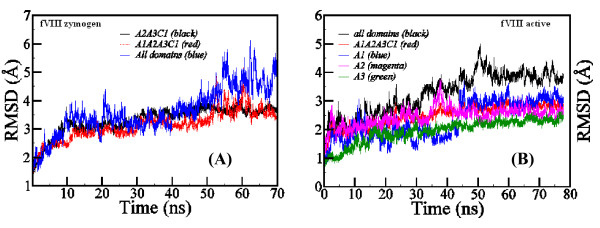
**The RMS deviations of the backbone atoms of A) the zymogen (left) and B) the activated form (right) of factor VIII as compared to the starting structure**.

**Figure 5:**
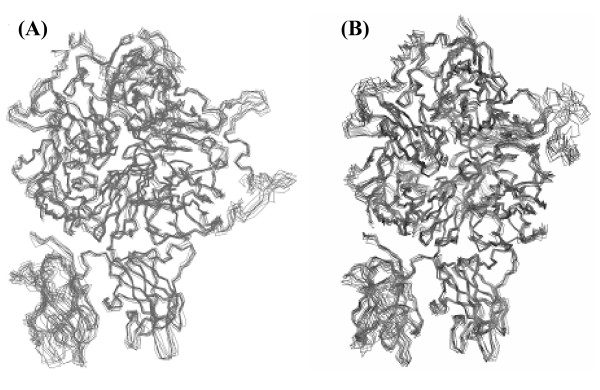
**Backbone superimposed structures of A) zymogenic (left) and B) activated (right) forms of factor VIII, representing the top ten clusters of the MD trajectory**. The last 10 ns of the simulation data (70-80 ns) was used for clustering.

In order to understand the overall dynamics of the structures, we monitored the structural changes over the simulation time by computing the RMS deviations with reference to the starting structure (Figures 4a-b). The RMS deviations of fVIII were much larger when we considered all the five domains, but relatively stable when plotted the A1-A1-A3-C1 domains combined excluding the C2 domain. This difference can be attributed to the flexibility of the C2-domain than the other four domains. Despite the large binding surface between the C2 and the adjacent C1 domain, the two domains do not have energetically significant inter-domain interactions between them (vide infra). We have generated 10 possible models of fVIII and fVIIIa by clustering the last 10 ns of the MD trajectory. The backbone superimposed structures of the 10 models for both forms are presented in Figure 5. It is evident from the figure that while the A1-A2-A3-C1 domains appeared relatively stable, the C2-domain showed significant flexibility in both the structures. Also, it is evident from the figure that the A2-A3 peptide linker region showed some degree of flexibility in the zymogenic form. The C-terminus of the A2-domain in the activated form that is mostly occupied by negatively charged residues also showed large flexibility.

**Figure 6:**
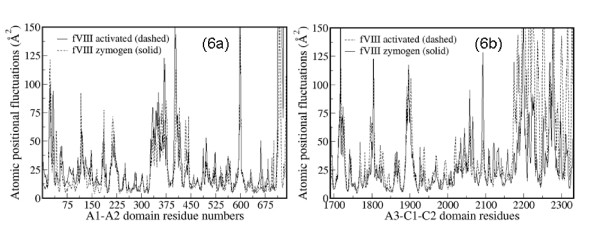
**Atomic positional fluctuations in A1-A2 and A3-C1-C2 domains of A) active and B) zymogenic forms of fVIII**. The data corresponds to 2 ns of MD simulation data extracted from 75 to 77 ns.

To probe the intra and inter-domain mobility of the solvent-exposed loop regions further, we computed the atomic positional fluctuations in fVIII and fVIIIa models. A comparative plot of the positional fluctuations averaged over the last 2 ns of MD trajectory is shown for the A1-A2 and A3-C1-C2 domains in Figures 6a,b. Atomic positional fluctuations are often used to characterize the domain flexibility in multi-domain protein systems as well as to understand the hyper-variability of the solvent exposed loop regions. Typically, the loops that have positional fluctuations more than 50 Å^2 ^may be considered hyper-variable in the protein structures. In experimental X-ray crystal structures, the B-factors usually provide such information. While it is difficult to quantitatively relate the positional fluctuations from MD simulations with that of experimental B-factors in the X-ray crystal structures, both share a qualitative comparison. Since the published X-ray crystal structure of fVIII was derived by fitting the homology models, the crystallographic B-factors were assigned >190 Å^2 ^of positional uncertainty. Consequently we could not make direct comparison with the computed atomic positional fluctuations. It is evident from Figures 6a,b that the overall flexibility of the solvent-exposed loop regions is very similar in both zymogen and activated forms. In line with the RMSD plot (Figure 4a), the conformational flexibility of the C2 domain (from Cys2021 to Cys2326) is clearly evident in both fVIII and fVIIIa with more than 125 Å^2 ^of the atomic positional fluctuations. The acidic linker peptide region between the A1 and A2 domains spanning the residues Pro330 and Lys380, the loop regions 21-34, 397-415, 594-600, 1713-1725, 1796-1805 and 1889-1901 exhibited relatively large mobility during the MD simulations.

## Discussion

### Structure and conformation of A1/A2 and A2/A3 linker peptides

**Figure 7:**
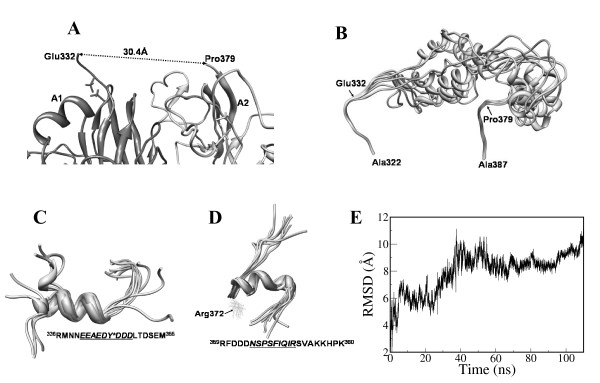
**Models used for generation of linker peptide between A1 and A2 domains**, A) The A1 and A2 domains with missing linker peptide and the distance between the core residues Glu332 (A1 domain) and Pro379 (A2 domain) is shown. B) The top ten structures of the proposed linker peptide derived from clustering the last 30 ns of 100 ns MD trajectory are presented. Converged structures of isolated sequences C) Arg336-Met355 and D) Asn369-Lys380 in the linker peptide (based on 50 ns of MD simulations each) to demonstrate the reproducibility of the helical nature of the sequences is shown. E) The RMS deviations in the backbone (Cα) atoms during the simulations of the linker peptide model with reference to the starting structure are shown.

In the present study, the acidic-rich linker peptide connecting the residues Cys329 of A1-domain and Thr381 of A2-domain was derived based on MD simulations for a total time of 200 ns. Though the starting structure for the loop optimization was based on a linear conformation, over the MD refinement, the linker peptide adopted helical structure for two acidic rich regions. The helices correspond to the sequence ED**Y**^**sulf**^DDDLTD from Glu344 to Asp352 and the activation peptide sequence SFIQIR from Ser367 to Arg372 as shown in Figure 7. The linker peptide conformation was stabilized, as may be seen from RMS plot (Figure 7e), with three ion-pair interactions with the A1 and A2 domains. These correspond to interactions of Glu332:Asp302; Arg336:Asp302/Glu331 and Arg359:Asp459. The two helices are connected to the A1 and A2 domains by largely flexible structure with no specific secondary structure. The predicted helical structure for Glu344-Asp352 may have a functional significance as this region was implicated to have a potential role in recognizing the substrate fX by the tenase complex (fVIIIa.fIXa). In order to verify that the helical preference for the two proposed regions between A1 and A2 domains is reproducible, we carried out two independent MD simulations on the peptides correspond to the residues Arg336-Met355 and Val359-Thr380 for 50 ns (data not shown). Both structures converged over the simulation period to helical conformation (Figures 7c,d). The reproducibility of the helical nature of the two proposed regions assures the reproducibility of the structure prediction for the two acidic patches.

**Figure 8:**
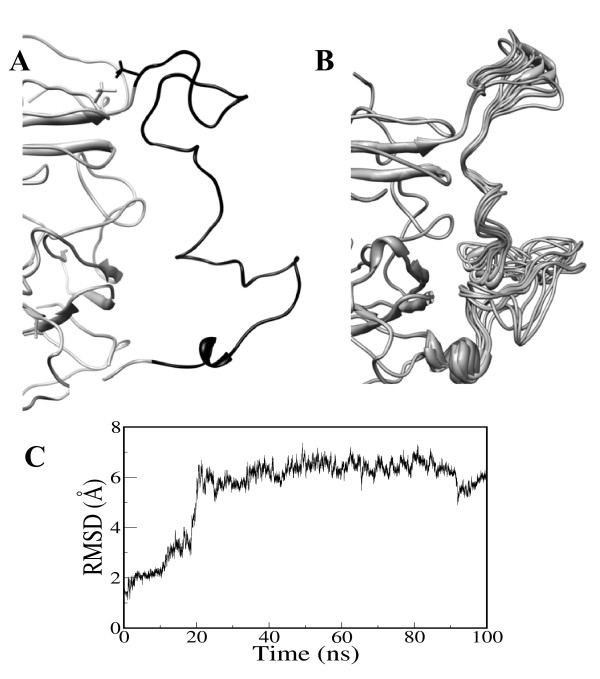
**The structural convergence of the A2-A3 linker peptide generated using a reduced model between A2 and A3 domains. **A) The starting conformation, B) the converged structures (based on clustering the last 30 ns of MD trajectory) of the linker peptide and C) the corresponding backbone (Cα atoms) RMS deviations with reference to the starting conformation are shown.

The linker peptide connecting Cys710 (A2-domain) and Thr1695 (A3-domain) in the B-domainless fVIII form was modeled based on a truncated model between the A2 and A3 domains as described in the modeling section. The residues at the C-terminus end of A2 domain are predominantly acidic with three tyrosine residues at positions 718, 719 and 723 that were post-translationally sulfated. In addition, the residue 1680 at the junction of A2-A3 domain is also sulfated. The A2-A3 linker peptide was stabilized over the 100 ns of MD simulations as shown in the RMSD plot (Figure 8c). The last 20 ns of the MD trajectories were clustered into 10 conformations and are shown in Figure 8b. It is evident from the figure that the optimized loop structure shows significant flexibility with the acidic patch of residues at the C-terminus of the A2-domain form a stable hairpin-like structure with the sulfated tyrosines projecting out to the surface.

### Structural features of metal ions in factor VIII/VIIIa

The function and stability of the fVIII depend critically on metal ions, particularly calcium and copper ions, covalently bound to the protein. Studies based on the reconstitution of isolated subunits as well as site-specific mutagenesis studies indicated that Type-I (Cu^+^) and Type-II (Cu^+2^) ions covalently bind to factor VIII. These studies suggested that the primary role for copper (Cu^+^) was to enhance the inter-chain affinity by ~100 fold [16]. It was also showed that the Type-I copper (Cu^+1^) bound to the inter-domain interface is not sufficient alone to regenerate the full co-factor activity but might need a calcium ion binding to augment the stability of the structure. However, the precise location of the calcium ion binding, probably a low-affinity binding site as suggested by Wakabayashi *et al*, is not yet experimentally characterized [17]. It is also interesting that the calcium ion, itself, has little effect on inter-subunit affinity yet converts the inactive dimer to an active form [18]. While high (mM) concentrations of Cu(II) fail to support fVIII reconstitution, low (μM) levels of Cu(I) or Cu(II) stimulate reconstitution in the presence of calcium or manganese ions [19]. Activity generation due to calcium ion binding to fVIII was shown to be a slow process and thus a slow conformational transition may be involved [20]. Results from Factor Xa generation experiments suggested that fVIII might possess two calcium-binding sites with differing binding affinities. A high affinity calcium ion was proposed to bind to A1-domain with K_d _value of 8.9-18.9 μM while a low-affinity site was proposed to bind to an unknown site with a weak K_d _value of 4.0 mM [21].

### Calcium-ion coordination

**Figure 9:**
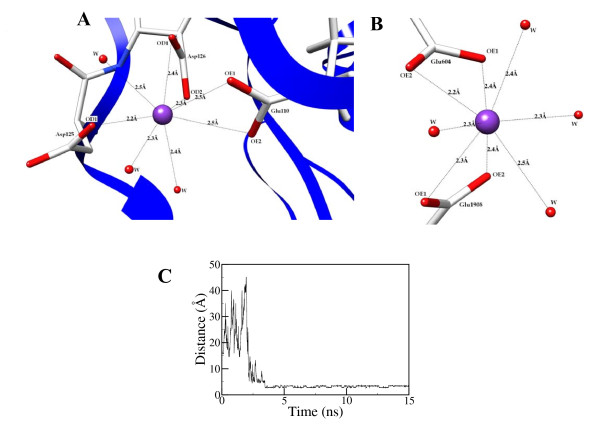
**The octahedral calcium coordination network in A) A1-domain; B) at the interface of A2 and A3 domains and C) the distance plot of a solvent calcium ion coordination with one the side-chain carboxylate atoms are shown**.

Several early mutagenesis studies have suggested that the A1-domain region spanning residues 110-126 constitute a calcium-ion binding site [22]. The loop has eight acidic residues (E110, E113, D115, D116, E122, E124, D125 and D126) providing an ideal site for calcium coordination. In the starting X-ray crystal structure, one calcium ion was located in the A1 domain with coordination to the side-chain oxygen atom of Asp126 and the backbone oxygen atom of Ala112. No other coordinating oxygen atoms were located within the 5 Å vicinity of the calcium atom. The calcium ion has been well characterized in several blood coagulation proteins to bind to three to four amino acids to maintain an octahedral coordination network [23,24]. Experimental alanine mutagenesis studies demonstrated that the mutation of Glu110, Asp116, Glu122, Asp125 or Asp126 led to significant reduction (or lost) in Ca^2+ ^binding affinity [25,26]. In contrast, the Ala-substitution at Glu113, Asp115 or Glu124 showed wild-type-like activity with little or no reduction in calcium ion affinity. To be consistent with the experimental data, we placed a calcium ion in the A1-domain loop with initial coordination optimally close to the side-chain carboxylate groups of all possible negatively charged residues (Glu110, Glu113, Asp125 and Asp126). Over the course of molecular dynamics simulation, the calcium ion developed a stable octahedral co-ordination network with the side-chain carboxylate group atoms OD1 and OD2 of Asp126, OE1, OE2 atoms of Glu110 and OD1 of Asp125. Three oxygen atoms from the solvent water were also involved in completing the coordination network (Figure 9a). The coordination network of calcium ion in the A1-domain is identical in both active and zymogenic forms of fVIII.

As mentioned previously, it was proposed in biochemical studies that fVIII might also have a second low-affinity calcium ion bound to the A-domains that contribute to the inter-domain affinity. While there was no structural data so far to characterize such interaction, the current MD simulations predicted, for the first time, a stable calcium ion binding to the interface of A2 and A3 domains. The simulation setup of fVIII and fVIIIa contained several calcium ions in the water solvent that were not bound to the protein. Within the initial 20 ns of the simulation period, one of the calcium ions from the solvent emerged to appear close to Glu604 of A2-domain and Glu1908 of A3-domain residues and developed a stable octahedral coordination network with the side-chain oxygen atoms (OE1 and OE2) of the carboxylate groups together with four oxygen atoms of the solvent water molecules (Figure 9b). While the structural and functional relevance of the second calcium ion is not known, the simulations suggest that this might be considered as the second low-affinity calcium-ion binding to the fVIII protein that involves A2 and A3 domains with Glu604 and Glu1908 residues. Perhaps mutation of either or both residues by experimental mutagenesis studies might further clarify the role of the second calcium ion in the proposed site.

### Copper ions coordination

**Figure 10:**
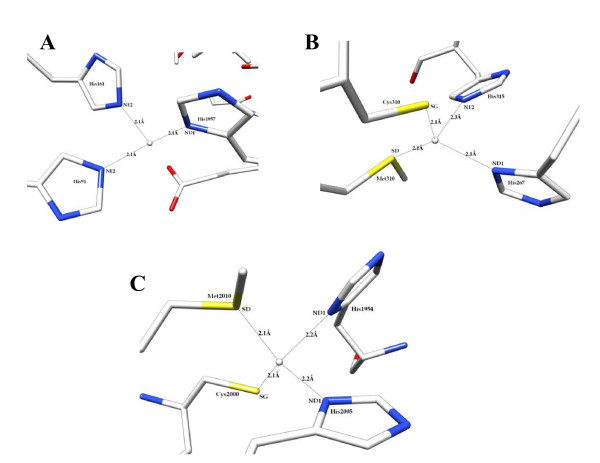
**The tetrahedral copper ion coordination network in A) the A1 domain, B) the A3 domain and C) at the interface of A1 and A3 domains are shown**.

Factor VIII is structurally homologous to the copper-binding human ceruloplasmin and factor Va [27,28]. The comparison of the three structures, together with the biophysical and experimental mutagenesis data [29,30], suggests that two Type-I (Cu^+1^) copper ions bind to the A1 and A3 domains. The reconstitution of fVIII from isolated heavy and light chains revealed a primary role for copper ion was to enhance the inter-chain affinity by ~100 fold [31]. It is known, in many copper binding proteins, that the copper ion coordinates with a network of residues comprising His/Cys/Met or with His/His/His network [32-34]. In factor VIII, two such ideal sites exist within the A1 and A3 domains. These sites are: His267/Cys310/His315/Met320 in A1-domain and His1954/Cys2000/Met2010/His2005 in A3-domain. Also, a His99/His161/His1957 network was found between A1 and A3 domains. The site-specific mutagenesis studies have indicated that the Cys310Ser mutation in the A1-domain showed marked reduction in the specific activity of fVIIIa. In contrast, the A3-domain mutations Cys2000Ser and His1957Ala did not affect factor VIII specific activity where as the A1-domain mutation His99Ala yielded ~75% reduction compared to the wild-type. While some of these studies provided a conflicting view of the role of copper ion co-ordination, it is noteworthy that the mutagenesis studies were based on single-point mutations. Within the A1 and A3 domains, the copper is coordinated with two nitrogen atoms of Histidines and side-chain sulfur atoms of Cys and Met residues. It is possible that the binding affinity might not be altered to noticeable effect by mutating single-residue, while the other three residues could be sufficient to hold the copper-coordination network. The coordination network of the copper ions with the MD derived model is shown in Figures 10a-c. The ions maintained a stable coordination network during the simulation period. The histidine ring nitrogen atoms of His99, His161 of the A1 domain and His1957 of the A3-domain are coordinated with copper ion with the bond distance of ~2.1 Å. The copper ions within the A1 and A3 domains coordinated with the side-chain sulfur atoms of Cys and Met residues with the coordination distance of 2.1 Å while the histidine nitrogen atoms are at 2.2 Å distance.

### Post-translational modification sites

The B-domainless FVIII contains five ASN-glycosylation (N-linked) sites. These sites are located in A1 (41 and 239), A2 (582) and A3 (1810) and C1 (2118) domains [35]. All the five sites were modified to N-GlnAc linked carbohydrate attachments. The solution-structure showed that except for Asn2118, all the modified asparagines residues are solvent exposed. The Asn2118 residue is somewhat buried in the deep cleft between the A3 and C1 domains.

There are six tyrosine sulfation sites in FVIII. These are located at the positions: 346 (A1 domain), 1664, 1680 (A3 domain) and at 718,719 and 723 in the C-terminus end of the A2 domain [36,37]. Except for Tyr1664 which is not present in our model (The N-terminus of A3 domain begins from Ser1669 in our fVIII model), rest of the tyrosine residues were present in surface-loop regions with complete exposure of the side-chains to the solvent. The negatively charged sulfated tyrosines were implicated to have direct influence on the thrombin activation rates of fVIII. For instance, the mutation Tyr346Phe, located within the A1-A2 acidic linker peptide, accelerated the rate of fVIII cleavage by thrombin [38]. In contrast, the mutation of Tyr346Cys results in CRM^+ ^hemophilia A [39]. This defect was characterized by a discrepancy in one-state and two-stage clotting assays and the pathological implications were due to reduced activation rates. In the model of fVIII zymogen, the Tyr346^sulf ^is located within the α-helix formed by the acidic-rich sequence EDY^sulf^DDDLTD (from Glu344 to Asp352). One direct impact of Tyr346^suf ^mutation to cysteine could be the reduction of overall acidic nature of the helix and possibly destabilizing the helix. It may also be seen from Figures 2 and 3 that the sulfated tyrosine residues at 719,719 and 723 together with aspartic acid residues at 712, 717, 720, 721 and 724 generate a high density of acidic cluster with the hairpin-like loop at the C-terminus of A2-domain. This region was shown in experimental studies as a likely thrombin exosite binding region during the proteolysis of Arg372-Ser373 and/or Arg740-Ser741 peptide bonds [10,40]

## Inter-domain interactions in fVIII/fVIIIa

The structural stability originating from the inter-domain interactions of fVIII/fVIIIa is mainly due to the metal ion dependent association and the inter-chain association due to residue-level interactions. Indeed, 80% of the thermodynamic stability of fVIII is attributed to the latter. Upon the generation of the fVIIIa followed by the cleavages at three proteolytic sites in B-domainless fVIII, the activated form has limited half-life since it goes through the spontaneous inactivation through A2 subunit dissociation from the other domains [41]. While the specific structural rationale is not clear, the interactions between A2 and other domains were believed to be weak and limited [42,43]. An accurate account of atomic level interactions is critical to understand the origins of stability as well as to provide a structural correlation to the known genetic or synthetic mutation data. The detailed interaction data among the A-domains of fVIII/fVIIIa is tabulated in Table 1based on the equilibrated structures derived from the MD simulation data. In order to estimate the binding surface between fVIII domains, we computed the solvent-accessible surface area (SASA) using NACCESS program [44] with a probe radius of 1.4 Å. We estimated that the total surface area of fVIII and fVIIIa are 69543 and 69106 Å^2 ^respectively. The buried surface area among the A-domains (fVIII/fVIIIa) is as follows: A1:A2 (4022/3354 Å^2^); A2:A3 (4329/3690 Å^2^) and A1:A3 (2709/2632 Å^2^).

**Table 1:** Interdomain hydrogen bonding interactions and corresponding distances (in Å) among A1 (1-330), A2 (381-740) and A3(1669-2019) domains of the zymogenic and activated forms of fVIII.

fVIII zymogen		Activated fVIII			
*A1:A2 interactions*					
R240 (NH1)	L491 (O)	3.1	R240 (NH1)	L491 (O)	2.8
T275 (OG1)	D519 (OD1)	2.9	T275 (OG1)	T481 (OG1)	2.9
R282 (NH1)	D525 (OD1)	3.2	R282 (NH1)	D525(OD1)	2.7
R282 (NH1)	D525 (OD2)	2.6	R282 (NH1)	D525 (OD2)	3.1
R282 (NH2)	D525 (OD1)	2.8	R282 (NH2)	D525 (OD2)	2.9
R484 (NH1)	E272 (OE2)	2.7	S285 (OG)	F673 (O)	2.6
R484 (NH2)	E272 (OE2)	3.2	D482 (N)	H274 (ND1)	3.0
R484 (NH2)	Q305 (O)	2.9	V483 (N)	G273 (O)	2.8
R490 (NH1)	E341 (OE1)	2.8	R484 (NH1)	E272 (O)	2.9
R490 (NH1)	E341 (OE2)	3.3	R484 (NH1)	E272 (OE1)	2.8
R490 (NH2)	E341 (OE2)	2.9	R489 (NE)	E272 (OE2)	2.9
K493 (N)	F38 (O)	2.9	R489 (NH2)	E272 (OE2)	2.8
K493 (NZ)	D318 (OD1)	2.8	K493 (N)	F38 (O)	3.1
K493 (NZ)	E321 (OE1)	2.7	K493 (NZ)	D318 (OD1)	3.5
K493 (NZ)	E321 (OE2)	3.3	K493 (NZ)	D318 (OD2)	2.9
K512 (NZ)	E354 (OE1)	2.8	K493 (NZ)	E321 (OE2)	2.8
Q645 (N)	S314 (OG)	3.3	S674 (OG)	E287 (OE2)	2.7
Q645 (NE2)	S313 (O)	3.5			
S674 (OG)	E287 (OE1)	2.6			
					
*A1:A3 interactions*					
R226 (NH1)	D1909 (OD2)	3.0	R220 (NH1)	E1914 (OE2)	2.8
R226 (NH2)	D1909 (OD1)	2.9	R220 (NH2)	E1914 (OE1)	2.8
R226 (NH2)	D1909 (OD2)	2.9	R220 (NH2)	E1914 (OE2)	3.3
R1719 (NH1)	D221 (OD2)	2.9	R226 (NH1)	D1909 (OD1)	2.9
R1719 (NH2)	D221 (OD1)	2.8	R226 (NH1)	D1909 (OD2)	3.2
R1997 (NH1)	E113 (O)	2.9	R226 (NH2)	D1909 (OD2)	2.7
R1997 (NH2)	E113 (O)	2.9	T263 (OG1)	E2004 (OE2)	2.7
K1972 (NZ)	D150 (OD1)	2.9	R1719 (NH1)	D221 (OD1)	3.1
K1972 (NZ)	D150 (OD2)	2.9	R1719 (NH1)	D221 (OD2)	2.7
N1977 (ND2)	S289 (OG)	2.9	R1719 (NH2)	D221 (OD1)	2.7
N1977 (ND2)	P290 (O)	2.8	R1997 (NH1)	E113 (O)	3.2
K1992 (NZ )	Y105 (O)	2.9	R1997 (NH2)	E113 (O)	2.8
			N1977 (ND2)	P290 (O)	
			K1992 (NZ)	Y105 (O)	2.8
					
*A2:A3 interactions*					
Y664 (N)	K1967 (O)	3.0	N414 (ND2)	Q1906 (OE1)	2.9
T669 (OG1)	P1980 (O)	3.1	Y664 (N)	K1967 (O)	2.9
S695 (OG)	D1842 (OD1)	2.9	S695 (OG)	D1842 (OD1)	2.8
Y729 (N)	E1794 (OE1)	2.8	S695 (OG)	D1842 (OD2)	3.5
Y729 (OH)	R1797 (O)	2.9	S732 (OG)	E1794 (OE2)	2.7
N734 (ND2)	E1793 (OE2)	2.7	K733 (NZ)	D1795 (OD1)	2.7
N735 (ND2)	D1795 (OD1)	2.8	R1803 (NH1)	P739 (O)	2.8
E738 (N)	E1671 (O)	3.0	R1803 (NH2)	E738 (OE2)	2.9
R740 (NH1)	E1811 (OE1)	2.8	R1803 (NH2)	P739 (O)	2.9
R740 (NH2)	E738 (OE1)	2.7	K1804 (NZ)	E738 (OE1)	2.7
R740 (NH2)	E738 (OE2)	3.4	K1833 (NZ)	Y664 (O)	2.8
S1669 (N)	E738 (O)	3.1	K1833 (NZ)	D666 (OD1)	2.8
S1669 (OG)	E738 (OE2)	2.7	N1904 (ND2)	N414 (O)	2.9
V 1670 (N)	P739 (O)	3.2	N1904 (ND2)	N414 (OD1)	3.0
E 1671 (N)	E738 (O)	3.1	Q1906 (N)	P606 (O)	3.0
D 1795 (N)	N734 (OD1)	3.1	K1967 (NZ)	E665 (OE1)	2.9
R 1797 (NH1)	L687 (O)	3.0			
T 1826 (N)	Y664 (OH)	3.1			
K 1833 (NZ)	Y664 (O)	2.9			
K 1833 (NZ)	D666 (OD1)	2.6			
R 1900 (N)	E607 (OE1)	2.7			
A 1901 (N)	E607 (OE1)	3.2			
Q 1906 (N)	P606 (O)	2.8			
Q 1906 (NE2)	E604 (OE1)	2.9			
K 1967 (NZ)	E665 (OE1)	2.8			

### A1-A2 binding surface

Analysis of the interaction data showed significant contribution form several ion-pair interactions that promote stability between A1 and A2 domains as shown in Table 1. Some key interactions include the ion-pairs: Arg282:Asp525; Arg484:Glu272; Arg490:Glu341; Lys493:Asp318/Glu321 and Lys512:Glu354. Apart from these major contributions, several hydrogen bond interactions were identified at the binding surface between A1 and A2 domains. It is worth-mentioning that the interaction data from present MD models correlate well with the published experimental mutagenesis data. In a recently study on the characterization of A-domain residues that contribute to the fVIII/fVIIIa stability, Wakabayashi and Fay investigated a panel of mutations that contribute to the A2-domain affinity with A1 and A3 domains [6]. They concluded from the study that the decay of fVIII stability depends on the specific residues in the decreasing order of: Arg282 > Ser524 > Asn684 > Arg531 > Ser650 > Glu1829 > Tyr664 > His281. These mutations showed marked decrease in the protein stability relative to the wild-type fVIII in a one-stage clotting assay and two-stage chromogenic factor Xa generation assay. The design of these experiments was based on a homology model of A1-A2-A3 domains derived from human ceruloplasmin template that shares ~35% sequence identity with fVIII. Comparison of our MD model with the homology model showed significant differences in the inter-domain surface and perhaps the effect of some of the proposed mutations could be due to the direct/indirect influence of some of the neighboring residues than the specific mutations proposed in their experimental work.

The mutations of Arg282, Ser524 and Arg531 were shown to increase the fVIII decay by > 5-fold. As shown in Table 1, the Arg282 is ion-paired with Asp525 and the Arg282Ala mutation would impact the binding interactions between A1 and A2 domains. While the effect of Asp525 mutation was not studied, the neighboring residue Ser524 is located in the same loop that Asp525 resides. The Ser524Ala mutation was also shown to significantly increase the fVIII decay. This residue is solvent exposed in the model of fVIII and perhaps alanine mutation would effect the loop conformation that might, in turn, alter the conformation of Asp525 and consequently the ion-pair interaction with Arg282. It is also worth mentioning the ion-pair interaction between Arg531 and Asp519. The Arg531Ala mutation was shown to affect the inter-domain affinity. While this residue is not involved in the inter-domain interactions, it forms ion-pair with Asp519 which is located in the same loop segment that Arg525 is located. Thus, the Arg531 mutation would impair interactions with Asp519 that might have structural consequence in affecting the stability of Arg285:Asp525 interactions.

### A1-A3 binding surface

Despite the relatively smaller binding surface compared to that of A1:A2 domains, the A1 and A3 domains are largely stabilized by the copper ion coordination at the A1 and A3 domain interface. In addition, four major ion-pair interactions add to the overall binding affinity between the two domains. These are Arg220:Glu1914; Arg221:Arg1719; Arg226:Asp1909 and Glu113:Arg1997. It should be noted that the Glu113 is located in the calcium-binding loop within the A1 domain. The mutagenesis studies suggested that the Glu113Ala mutation within this calcium binding loop showed four-fold increase in co-factor activity. While the mutation of Glu113 to large polar residues was shown to be detrimental to the co-factor activity, Glu113Ala mutation seems to increase the stability. In our structure, we identify that the backbone oxygen atom of Glu113 forms hydrogen-bonding with two hydrogen atoms of the side-chain of Arg1997 within the A3-domain. We observed that this interaction was consistently maintained over the entire stabilized MD trajectory and, indeed, was the only inter-domain interaction that contribute to the association of the calcium binding loop to the A3-domain. Therefore, the mutation of Glu113 into other bulky groups might disrupt this interaction and could be deleterious to the inter-domain affinity between A1 and A3 domains.

### A2-A3 binding surface

The interactions between the A2 and A3 domains in fVIII zymogen are governed by a large number of hydrogen bonded interactions. The A2:A3 surface has three ion-pair interactions due to the clustered network of charged residues Arg740:Glu378/Glu1811; Lys1833:Asp666/Tyr664; Glu607:Arg1900/Ala1901. Unlike the A1:A2 and A1:A3 domains that did not show a great loss of inter-domain interactions upon activation, the A2:A3 domain interface appears to loose a large number of hydrogen bonds (9 out of 25) as seen from the Table 1. These differences reflected on the corresponding loss of 640 Å^2 ^surface area in the activated form when compared with that of the zymogen. The A2-subunit dissociation in the activated form and the reduced half-life in the blood plasma could be attributed, at least in part, to the diminished interactions between the A2 and A3 domains. In a study based on the Glu1829Ala mutation, the A3-domain residue Glu1829 was proposed to contribute to the A2 subunit retention [9]. In the zymogen structure, we noticed that the residue was bonded to the backbone oxygen atom of Arg1966 of A3-domain. A similar observation can be seen in the reported X-ray crystal structure of fVIII [14]. However, Glu1829 is located in the loop that has direct interactions with the A2-domain interface. At the interface, Tyr664 of A2-domain is observed to have hydrophobic interactions with Phe1830 residue of A3-domain via π-π stacking. Thus, the mutation of Glu1829 could be seen as the neighbor-effect in reducing the A2:A3 domain interactions. The Asp666Ala mutation was shown to increase the fVIII decay rates [45]. As shown in Table 1, Asp666 residue is bonded to Lys1833 though ion-pair.

### Interactions of C1-C2 domains with A1-A2-A3 domains

The platelet binding C-domains together share 16962 Å^2 ^of buried surface with the A1/A2/A3 domains. Despite the large binding surface, the C-domains have limited stabilizing interactions with the A-domains. We noticed that only one clustered ion-pair formation of Glu1751 with Arg2116 and Tyr2105. Other H-bonding interactions include Arg4:Tyr2332; Ala1866:Ser2119; Glu1754:Gln2113; Arg2116:Thr2122 and Tyr2115:Pro2142. Interestingly, the C1-domain shares 670 Å^2 ^of surface area with C2-domain but no hydrophobic or electrostatic interactions were found between the two domains. As discussed in the early part of the text, the large-scale domain motions, that we observed (from RMSD calculations) when all the domains are considered, stems for the lack of stabilizing interactions between the C2 and C1 domains. As the C1-C2 domains seem to bind to the platelet surface together, the overall structure of fVIIIa might adopt a stabilizing orientation, for functional co-factor activity, on the anionic phospholipids surface through the likely hydrophobic interactions with C1-C2 domain residues Phe2093, Leu2251, Leu2252 and Met2199 residues. All these residues are projected towards the possible membrane surface.

### Structural differences between zymogenic and activated forms

The B-domainless factor VIII zymogen has three proteolytic sites at Arg372 (A1-A2 junction), Arg740 (A2-B junction) and Arg1689 (at the N-terminus of A3) that must be cleaved by the physiological activator, thrombin [46,47]. While the precise order of the cleavages is not well-known, the proteolysis of Arg372-Ser373 peptide bond is believed to be penultimate step in the activation process of the zymogen and is essential for full co-factor activity of fVIIIa. Recent biochemical studies suggested that the proteolysis at either Arg740 or Arg1689 facilitates subsequent bond cleavages during the thrombin catalyzed activation [48]. The Arg372-Ser373 site is located in the linker peptide that has several acidic residues between the A1 and A2 domains. While the structural rationale behind the essential pre-requisite of the cleavage of Arg372-Ser373 peptide bond is not known, it was hypothesized that the cleavage of the bond exposes a cryptic functional factor IXa-interactive site in the A2-domain [49]. The acidic linker peptide conformation in the solvent-equilibrated zymogen model adopts the conformation that blocks the Tyr555-Asp569 loop in the A2 domain (highlighted in Figure 2). The Arg372-Ser373 cleavage site can be clearly seen to mask the fIXa binding site in the zymogen model. In the factor X activating complex fIXa/fVIIIa, residues 555-569 of the A2 domain are thought to bind to the protease domain of factor IXa [50]. In another study, it was also proposed that helix 330-338 of fIXa interacts with 555-569 loop of A2-domain [51]. Taken the data together, it becomes apparent from the current zymogen model that the activation peptide loop between the A1 and A2 domains blocks the complex formation between fVIII and fIXa. Based on MD simulations on fVIIIa, in which the Arg372-Ser373 bond gets cleaved, our model of fVIIIa predicts that the cleaved peptide linker between A1 and A2 domains undergoes significant conformational changes upon activation. Indeed the peptide segment Pro330-Arg372, in the activated form, completely restructures and relocates to the A1-domain (Figure 3). This conformational reorganization leads to full exposure of the putative fIXa binding site within the A2-domain for productive complex formation between the fIXa enzyme and fVIIIa co-factor. We must emphasize that the proposed linker peptide between the A1 and A2 domains is truly predictive in nature, albeit sheds some light on the likely conformation that the peptide linker might adopt. Whether the proposed conformation for the linker structure is indeed true or not can only be attested by experimental validation. Considering that we were able to reproduce the helical nature for the 9 residue long acidic patch and the short peptide preceding the proteolytic site may instill some confidence in the proposed structure.

## Conclusions

In the present study, we applied a step-wise modeling and MD refinement approach to develop the solution structural models of the zymogenic and activated forms of B-domainless human factor VIII based on the X-ray crystal structure of human fVIII. The models showed significant improvement in the stereochemical quality of the core region of the domains over the X-ray crystal structure. The solvent equilibrated models derived from several hundred nanoseconds of MD simulations in explicit water provided a detailed understanding of the dynamics of the multi-domain assembly. Structural defects in fVIII lead to the hereditary bleeding disorder, Hemophilia-A. More than 200 natural mutations are known that cause mild to severe bleeding in hemophilia patients. While a detailed structure-function correlation of the known genetic and synthetic mutations with fVIII/fVIIIa structures is beyond the scope of the present work, we were able to successfully correlate some of the recently reported experimental mutagenesis data with the MD derived models.

In the absence of experimental structural data, we applied MD simulation techniques to predict the likely conformations for the linker peptides between the A1:A2 and A2:A3 domains. Though the models are derived from long time scale MD simulations and appear to be well-stabilized during the dynamics, we must emphasize that the loop conformations are truly predictive in nature and need experimental attestation. However the proposed model for the linker peptide between A1 and A2 domains may shed some light on the current understanding of the zymogenicity of fVIII. The linker peptide between A1 and A2 domains was predicted to block the "steric access" of the putative factor IXa binding region (Tyr555-Asp569) in the A2-domain. The simulation of the activated form predicted that the loop re-organizes upon cleavage and relocates to A1 domain, thereby exposing the Tyr555-Asp569 loop and the surrounding region. Since this loop was proposed by several experimental studies as functionally important for fIXa enzyme binding, the conformational differences between fVIII and fVIIIa in the current study may provide a structural basis for the zymogenecity of fVIII and the importance of the proteolysis of Arg372-Ser373 peptide bond for productive interactions between fIXa and fVIIIa. The extensive MD simulations employed in the current study also characterize the specific residues involved in the co-ordination network of the high-affinity calcium-ion within the A1-domain. The proposed data related to the inter-domain interactions may serve as a reasonable structural basis to design the site-specific mutagenesis studies to further characterize the residues involved in promoting the overall stability of fVIII/fVIIIa and, in turn, the validation of the proposed solution structures.

## Methods

Upon the loss of a 19 residue length signal peptide, the mature fVIII zymogen circulates in blood as a single chain protein with 2332 residues (A1-A2-B-A3-C1-C2) out of which the B-domain consists of 573-residues [52]. Factor VIII was experimentally characterized to possess eight disulfide bonds [53]. Each of the A-domains contains two disulfide bonds and one free cysteine residue while the C1 and C2 domains possess one disulfide bond in each domain. The function of fVIII has long been implicated to depend critically on the binding of calcium and copper ions to the protein [29,54-57]. Several biochemical studies proposed that the A1 domain contains one high-affinity calcium ion while there might be a low-affinity calcium ion bound to the other A-domains, though the specific details were not known [58,59]. Also, the binding of a Type-I copper ion in each of the A1 and A3 domains and a Type-II copper ion binding at the interface of A1 and A3 domains were proposed based on comparison with copper ion binding network in several copper-binding proteins as well as experimental mutagenesis studies [60].

The B-domain in fVIII has no known structural homology in the protein database. Consequently, the modeling of B-domain is intractable to modern computational methods. The single-chain fVIII protein without the B-domain (741-1668) appeared to retain the full fVIII functionality as investigated by Donath *et al *[61]. In their study, the A1-A2 domains (Ala1-Arg740) were directly fused with A3-C1-C2 domains (Ser1669-Tyr2332) to generate a single-chain B-domainless fVIII protein. The fusion site, Arg740-Ser1669, was proposed to be functionally similar to other cleavage sites and was readily cleaved by thrombin and fXa. Thus, the exclusion of B-domain of zymogen fVIII may be considered to bear no effect on the structure-function studies related to fVIII protein and its activation pathways. The lack of experimental structural data for the missing linker peptides between A1:A1 and A2:A3 poses a major challenge to computational modeling as the peptide sequences are 40-50 residue long. In this study, we applied a step-wise modeling approach to generate the full models of fVIII and fVIIIa in several stages as described below.

### Step1. Refinement of the core structure of fVIII zymogen

The full model of single chain B-domainless fVIII was generated using the 3.7 Å resolution X-ray structure of the fVIII zymogen (PDB:2R7E) [13]. The crystal structural coordinates for the B-domainless protein reported include: Ala1-Asn214; Ala222-Gln334; Phe360:Asp725; Arg1689-Tyr2332. We modeled the missing loop residues between Asn214 and Ala222 using the MODLOOP server http://modbase.compbio.ucsf.edu/modloop/modloop.html[62]. The initial X-ray crystal structure was processed to include one calcium ion in the A1-domain and one copper (I) ion each in the A1 and A3 domains. The coordinates for the ions corresponded to those in the crystal structure. The calcium ion in the crystal structure was not well positioned as it was found to coordinate with only two atoms. These atoms correspond to one of the side-chain carboxylate atoms of Asp126 and the backbone oxygen atom of Ala110 within the A1-domain. The other likely coordinating atoms in the acidic-rich calcium binding loop were positioned at least 5 Å away from the calcium ion. The copper (I) ions within the A1 and A3-domain were located within the coordinating distance of His1954/Cys2000/His2005/Met2010 and His267/Cys310/Met320/His315. In addition, we introduced one copper (II) ion at the A1-A3 domain interface with the coordinating residues His99/His161 of A1- domain and His1957 of A3-domain, in consensus with the reported experimental studies as well as the structural comparison with other copper-binding proteins [29,63-66]. The starting model used for MD refinement contained the following residues: A1 (Ala1-Gln334); A2 (Lys376-Thr715); A3-C1-C2 (Arg1689-Tyr2332). The crystal structure together with the copper and calcium ions was immersed in a period box of waters to set up the MD simulation. The model was subjected to initial refinement of 15 ns of MD simulations in explicit water medium in order to relax the structure and to improve the overall stereochemical space of the structure. Analysis of the structure corresponds to the 15 ns of MD trajectory showed ~7 Å RMS deviation from the starting structure when superimposed the backbone atoms of the two structures. The resulting structure was subsequently used to model the missing linker peptides connecting the A1/A2 and A2/A3 domains as described below.

### Step2: Generation of acidic linker structure between A1 and A2 domains

The 46-residue length linker peptide between Glu332 and Pro379 is predominantly acidic with 18 residues being negatively charged. The sequence from 341 to 349 (EEAEDY*DDD) has large density of acidic residues. The residue Tyr346 (Y*) in the sequence represents sulfated tyrosine. Attempts to generate a possible structure for the linker peptide using homology modeling methods failed due to no corresponding template structure in the PDB. Consequently, the structure of the peptide was modeled using MD simulations for a total time period of more than 200 ns. Initially two beta-sheets that connect the linker peptide from the core residues of A1 (Ala322-Glu332) and A2 domain (Pro379-Ala388) were selected from the solvent equilibrated model (step1) as shown in Figure 7a. Analysis of the structure showed that the distance of backbone Cα atoms between Glu332 and Pro379 residues was 30.4 Å. A possible structure for the missing linker peptide between two connecting residues was generated by starting from a linear conformation for the sequence Glu332-Pro379. In order to let the structure fold into stable conformation, the structure was subjected initially to a 100 ns of unconstrained MD simulations in explicit water. Then, while imposing distance constraint of 30 Å between the two Cα atoms of Glu332 and Pro379, the structure was further simulated for additional 50 ns. The resulting linker peptide structure was then superimposed manually with the coordinates of the two beta sheet structures such that Glu332 and Lys377 residues form peptide bonds with Pro333 and His378 respectively. The conformation of the peptide bonds Glu332-Pro333 and Lys377-His378 were adjusted to acceptable Phi-Psi values. While constraining the residues Ala322-Cys329 of A1-domain and Thr381-Ala388 of A2-domain, the resulting model was subjected to 100 ns of MD simulations in explicit water. These residues were constrained in order to be able to insert, subsequently, the optimized loop structure back into the model of fVIII from step1. The last 30 ns of the stabilized MD trajectories were clustered to generate 10 possible conformations for the linker peptide as shown in Figure 7b. All conformations were superimposed against the MD equilibrated model of fVIII from step1. Since the two beta-sheets of A1 and A2 domains were constrained during the optimization of the linker peptide, we were able to align the coordinates of the beta-sheets with the corresponding residues in the MD equilibrated model (from step1). Out of the ten structures, only two conformations satisfied the spatial constraints such that the peptide could be inserted, with no steric clashes, into the model of fVIII. The rest of the structures did not fit into the fVIII structure as they overlapped with the core residues of A1 and A2 domains. We chose one of the two conformations to fit between the A1 and A2 domains to complete the missing linker peptide.

### Step3: Generation of linker peptide structure between A2 and A3 domains

The structure prediction for the linker peptide between the A2 and A3 domains followed a similar approach adopted in step2. MD simulations on fVIII model from step1 revealed that the C-terminal residues from 712 to 725 within the A2-domain appear to be flexible with no stabilization of the conformation. Consequently, these residues were deleted from the model of fVIII and reconstructed to build the linker peptide between Cys711 and Arg1689. The linker peptide from Cys711 to Arg740 of A2-domain and Ser1669-Arg1689 forms a contiguous sequence in the B-domainless fVIII model considered in the present study. The Cα-Cα distance between Cys711 (A2-domain) and Ser1690 (A3-domain) in the solvent equilibrated model of fVIII (from step1) is ~43 Å. In order to generate a possible structure for the linker peptide using MD simulations, a short model of A2 and A3 domains was selected from the MD equilibrated model of fVIII (from step1). The model comprised of selected residues of the A2 and A3 domains. The residues corresponded to: Val621-Ile639/Ser650-His660/Val678-Asp712 of A2-domain and Ser1690-Arg1705/Gly1760-Pro1809/Glu1811-Lys1827 of A3-domain. We chose the reduced model in place of the full structure of fVIII to increase the computational speed of MD simulations and also to minimize the computational cost. Initially, a random conformation was created for the missing linker peptide corresponding to the residues between Asp712-Arg740 and Ser1669-Ser1690 as a contiguous sequence (Figure 8a). The Arg740 (A2-domain)-Ser1669 (A3-domain) residues were directly bonded to represent the contiguous B-domainless fVIII structure. The tyrosine residues at positions 718, 719 and 723 were post-translationally modified in fVIII. Accordingly, the side-chain hydroxyl group in the three residues was modified to include the sulfated group. The positional constraints were applied during the simulation such that only the linker peptide region (Asp712-Arg740-Ser1669-Ser1690) moved while the rest of the protein remains constrained. The model was subsequently refined for 100 ns of MD simulation to generate a possible conformation for the linker peptide.

### Step4: Full model of B-domainless Factor VIII zymogen

The solvent-equilibrated models of A1-A2 and A2-A3 linker peptide structures, derived from the steps 2 and 3, were inserted into the equilibrated model from step-1 to generate the full structure of fVIII. Since the non-loop regions of the models used for A1-A2 and A2-A3 linker peptide modeling were constrained during the MD simulations, both models superimposed with the solvent equilibrated model (step1) with no steric clashes. Sequence characterization studies showed that asparagine (ASN) residues at positions 41, 239, 582, 1810 and 2118 were post-translational glycosylation sites [67]. Accordingly, these sites were modified to include the N-linked acetyl glucosamine (N-GlcNAc) carbohydrate attachments using the GLYCAM server http://glycam.ccrc.uga.edu/. In addition, tyrosine (TYR) residues at positions 346 (within the A1-A2 linker region) was modified to incorporate the sulfated tyrosines [38]. The other three tyrosine sulfation sites at 718, 719 and 723 positions at the C-terminus of A2-domain were already modified in the step3. The final model was subsequently refined for 70 ns of MD simulations in explicit water medium.

### Step5: Full model of activated Factor VIII

The domain organization of fVIII zymogen and its activated form, fVIIIa, are similar except that the activated form does not possess B-domain. The residues involved in the inter-domain interactions between A1:A2 and A2:A3 domains appeared to be comparable as suggested by recent site-specific mutagenesis studies [6,68,69]. In this study, a panel of 30 residues that appeared to be important for A1:A2 and A2:A3 interactions were subjected to alanine mutagenesis in both fVIII and fVIIIa. The reported protein decay rates in most of the mutants were comparable in both active and zymogen forms, suggesting that the residues involved in the inter-domain interactions might be identical. Thus, it may be reasonable to model the activated form from the zymogenic structure.

In order to build the activated fVIII model in which the Arg372-Ser373 peptide bond gets cleaved during the proteolysis by thrombin and/or Xa, the solvent-equilibrated fVIII zymogen structure, derived from the 70 ns of MD simulations, was modified to generate the activated form. This was accomplished by explicitly breaking the Arg372 and Ser373 bond and creating a hetero-trimeric complex among the A1 (Ala1-Arg372), A2 (Ser373-Arg740) and A3-C1-C2 (Ser1690-Tyr2332) domains. The full model of fVIIIa, thus obtained, was subjected to further refinement of 75 ns of MD simulations to obtain the solution structure of fVIIIa.

## MD simulations details

The MD simulations on all the structures, considered in the present study, were carried out in explicit water medium using AMBER10 program (Scripps Research Institute, San Deigo, CA) package with PARM99/SB force-field [70,71]. The force-field parameters for the copper ion were adopted from the earlier work [72]. The charge parameters for the sulfated tyrosine were derived by RESP-fit method on a model of Tyrosine sulfate using HF/6-31G* basis set to be consistent with PARM99 force-field parameters. The corresponding charges are: OG (-0.4318); S (1.2426); O1S (-0.64); O2S (-0.64); O3S (-0.64). The rest of the tyrosine residue charges were constrained during the charge fitting scheme.

The long-range interactions were treated using the particle mesh Ewald (PME) method [73]. Constant temperature and pressure (300 K/L.atm) were maintained throughout the simulations using the Berendsen scaling algorithm with coupling constant of 0.2 ps [74]. All bonds involving hydrogen atoms were constrained using the SHAKE algorithm. A time step of 2 fs was used to integrate the equations of motion. Before beginning the production-run simulations, the following equilibration protocol was followed.

1) The water molecules and counter-ions in the periodic box were energy minimized to a root mean square (RMS) gradient of 0.1 kcal/mol/A^2^. This followed a full minimization of the entire system.

2) NVT simulation for 5 ps was performed on the entire system.

3) NPT simulation for 50 ps was performed on the whole system except the backbone atoms of the protein residues to equilibrate the solvent waters and the protein side-chain atoms.

4) The entire system is subjected to slow heat-up from the initial temperature of 150 K to 300 K under NVT conditions.

5) Finally the system was subjected to production-run under NPT conditions and a constant temperature of 300 K. The final density of all the simulations in the current study was close to 0.99 g/cc.

Analysis and clustering of the MD trajectories were performed using the *ptraj *module of AMBER10 package. The simulations of full length structures of fVIII and fVIIIa were performed without any geometrical or positional constraints on the atoms. The computations were performed on the multi-core linux clusters using MPI/Infiniband version of PMEMD. The final setup of the zymogen and the activated forms of fVIII for MD simulations is given below.

a) Factor VIII: Total system size: 242,051 atoms; 1404 protein residues; 7 calcium ions (6 unbound to protein); 1 chloride ion; 73158 waters

b) Factor VIIIa: Total system size: 202,946 atoms; 1383 protein residues; 5 calcium ions (4 unbound to protein); 1 chloride ion; 60235 waters

## Structural coordinates information

The atomic coordinates of the solvent-equilibrated structures of fVIII and fVIIIa are deposited at protein model database http://mi.caspur.it/PMDB/main.php. The accession numbers for PDB coordinates is PM0076106 and PM0076119.

## Authors' contributions

DV planned and performed all the proposed work, structural analysis and wrote the manuscript.

## Supplementary Material

Additional file 1 **Figure S1**. The Phi-Psi Ramachandran plots (only non-glycine residues were considered) of the X-ray crystal (upper left), initial homology model (upper right), the solvent-equilibrated MD models of fVIII zymogen (lower left) and activated form (lower right). Click here for file

Additional file 2 **Figure S2**. Overlay of the MD equilibrated structure of fVIII zymogen (white) and the X-ray crystal structure (PDB:2R7E). The backbone atoms of the individual domains of A1, A2, A3, C1 and C2 of the X-ray crystal structures (color-coded) were superimposed against the MD model. Click here for file
