# Supplementary figures and images for: Structural investigation of zymogenic and activated forms of human blood coagulation factor VIII: a computational molecular dynamics study
Source: BMC Struct Biol. 2010 Feb 25;10:7. doi: 10.1186/1472-6807-10-7 (PMC2837666; doi:10.1186/1472-6807-10-7)

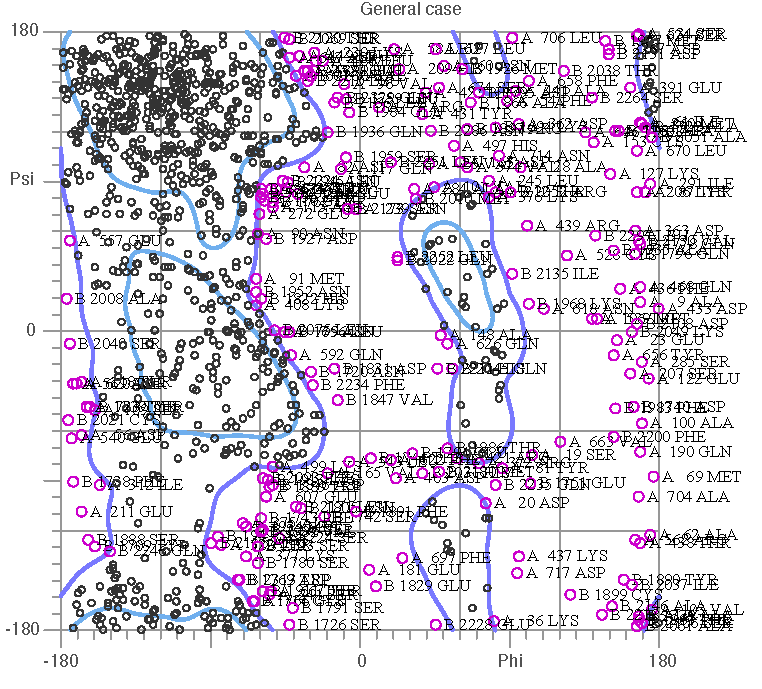

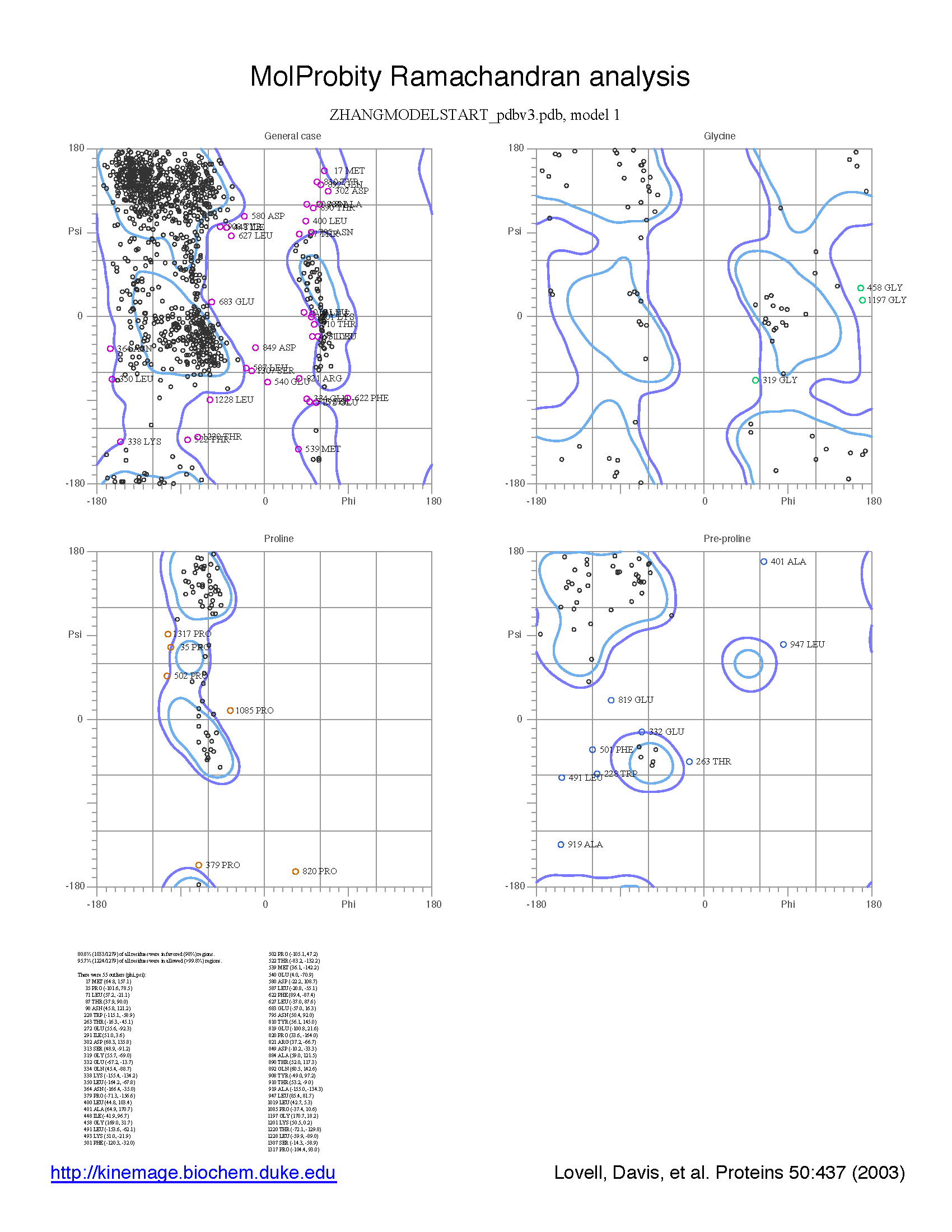


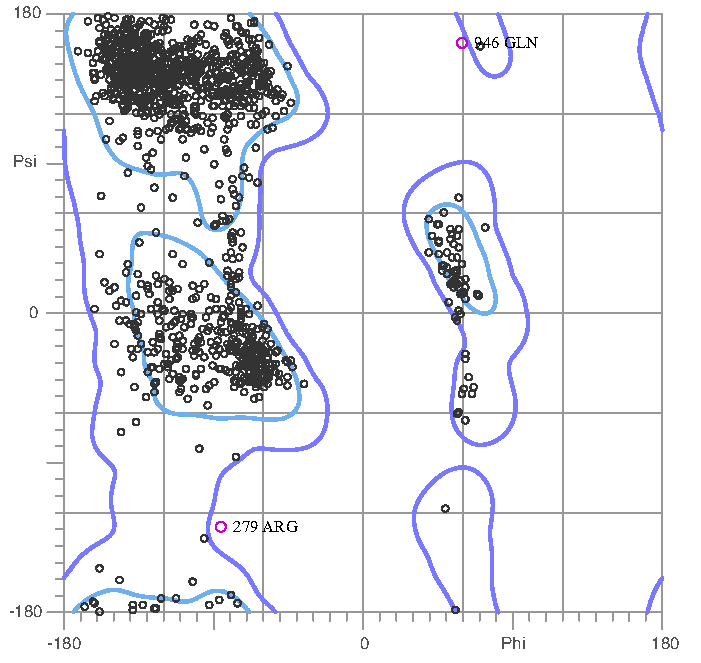

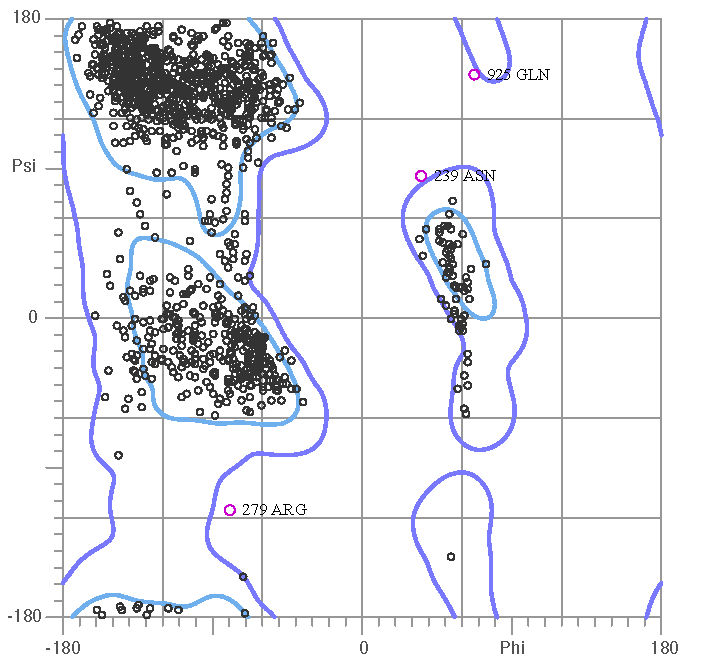

Supplement: Additional file 1 — Figure S1. The Phi-Psi Ramachandran plots (only non-glycine residues were considered) of the X-ray crystal (upper left), initial homology model (upper right), the solvent-equilibrated MD models of fVIII zymogen (lower left) and activated form (lower right). [file 1472-6807-10-7-S1.DOC]

**
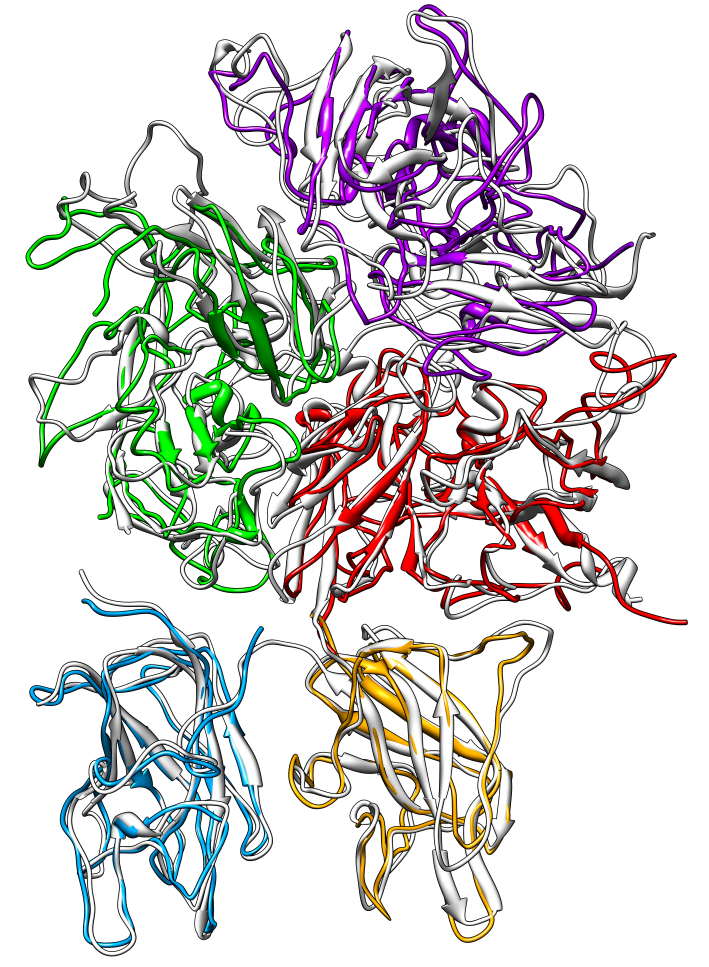
**

Supplement: Additional file 2 — Figure S2. Overlay of the MD equilibrated structure of fVIII zymogen (white) and the X-ray crystal structure (PDB:2R7E). The backbone atoms of the individual domains of A1, A2, A3, C1 and C2 of the X-ray crystal structures (color-coded) were superimposed against the MD model. [file 1472-6807-10-7-S2.DOC]
